# RACIAL AND ETHNIC DIFFERENCES HOSPITAL DISCHARGE DISPOSITIONS FOR OLDER ADULTS: A PUBLIC POLICY PERSPECTIVE

**DOI:** 10.1093/geroni/igad104.2855

**Published:** 2023-12-21

**Authors:** Sunny Kang

**Affiliations:** University of Baltimore, Baltimore, Maryland, United States

## Abstract

This paper presents two empirical studies regarding racial disparities in older individuals’ hospital discharge. The first study targeting factors associated with older Black patients’ readmission to ambulatory care, examined 7,619 older patients from the 2016 National Ambulatory Medical Care Survey; the second study focused on race and mental disorders’ impact on older patients’ nursing home (NH) admissions upon hospital discharge, analyzing 186,646 older patients from 2007 to 2010 National Hospital Discharge Survey. We mainly used Binary Logistic Regression Analyses (1) to examine the impact of racial disparities in ER readmission within one week to two months’ discharge and (2) to study the racial disparities in NH admissions upon hospital discharge. Based on the studies, policy efforts are called upon to address the specific needs of racial minorities using Medicaid as a primary payment method; as well as for individuals residing in facilities with higher concentrations of Medicaid beneficiaries. Discharge diagnoses such as lower-limb fractures, chronic ulcers, and mental health conditions, especially among Black individuals with mental health conditions increased the odds of NH admission. Other factors such as the Medicare as the primary payer for the hospitalization, being unmarried, and prior residence in a skilled nursing facility are also important predictors for NH admissions. Black individuals appear to increasingly be disproportionately represented in the NH population. This may relate in part to the greater morbidity they experience related to chronic conditions such as diabetes and heart disease, which warrants further investigation as to the reasons for their high presence.

